# Supplementary material for: Saccharomyces boulardii (CNCM I-745) improves intestinal damage in sepsis by remodeling intestinal flora structure
Source: Front Cell Infect Microbiol. 2025 Jul 31;15:1602792. doi: 10.3389/fcimb.2025.1602792 (PMC12350329; doi:10.3389/fcimb.2025.1602792)
Supplement: Supplementary file 1 [file DataSheet1.zip › summary/summary/7_Differential_analysis/2_LEfSe_result/Group_DW4_vs_DW0_vs_CW4_vs_CW0_vs_BW4_vs_BW0_vs_AW4_vs_AW0_0.05_3.0/LEfSe_Group_DW4_vs_DW0_vs_CW4_vs_CW0_vs_BW4_vs_BW0_vs_AW4_vs_AW0.barplot.pdf]

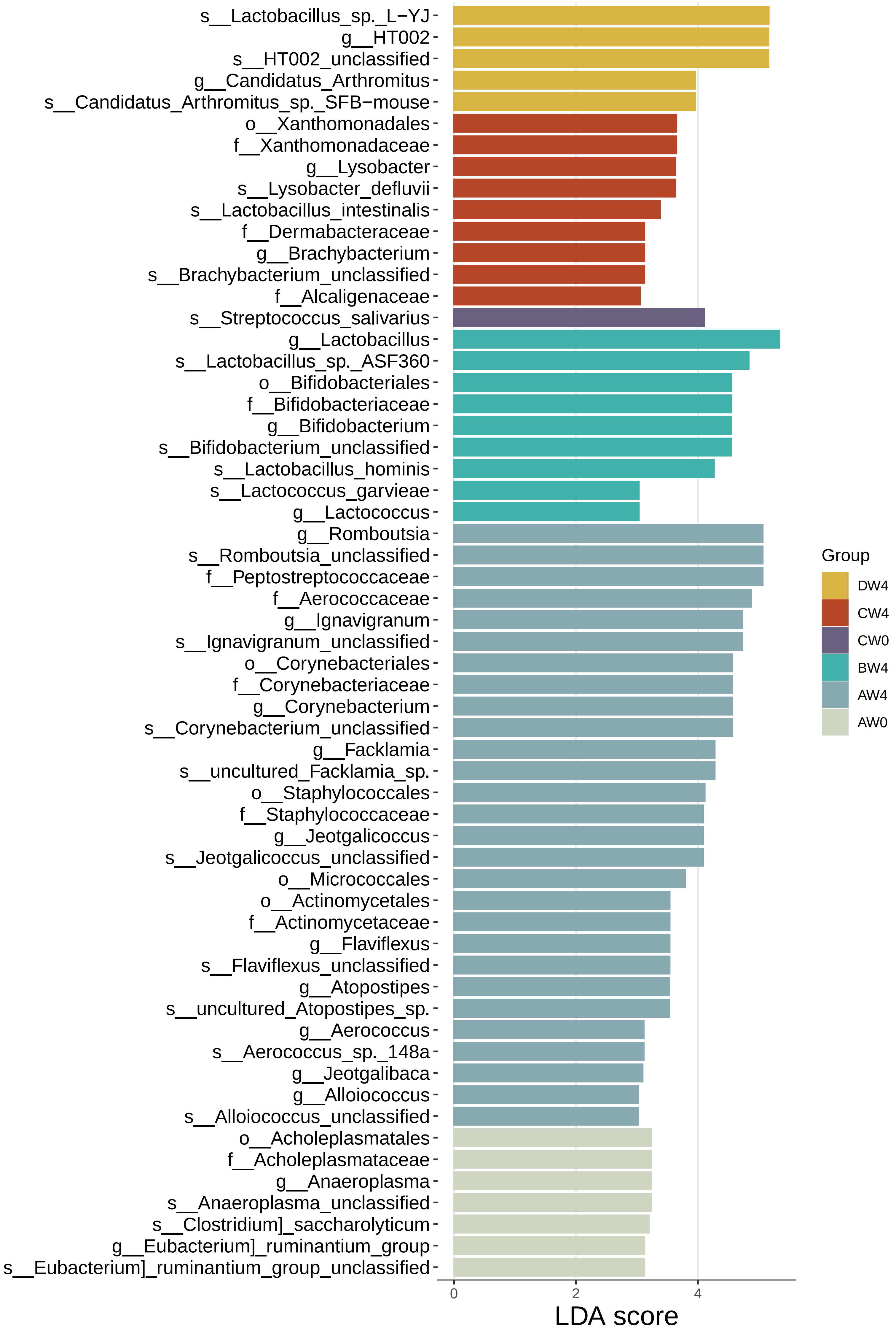

s__Lactobacillus_sp._L−YJ
g__HT002
s__HT002_unclassified
g__Candidatus_Arthromitus
s__Candidatus_Arthromitus_sp._SFB−mouse
o__Xanthomonadales
f__Xanthomonadaceae
g__Lysobacter
s__Lysobacter_defluvii
s__Lactobacillus_intestinalis
f__Dermabacteraceae
g__Brachybacterium
s__Brachybacterium_unclassified
f__Alcaligenaceae
s__Streptococcus_salivarius
g__Lactobacillus
s__Lactobacillus_sp._ASF360
o__Bifidobacteriales
f__Bifidobacteriaceae
g__Bifidobacterium
s__Bifidobacterium_unclassified
s__Lactobacillus_hominis
s__Lactococcus_garvieae
g__Lactococcus
g__Romboutsia
s__Romboutsia_unclassified
f__Peptostreptococcaceae
f__Aerococcaceae
g__Ignavigranum
s__Ignavigranum_unclassified
o__Corynebacteriales
f__Corynebacteriaceae
g__Corynebacterium
s__Corynebacterium_unclassified
g__Facklamia
s__uncultured_Facklamia_sp.
o__Staphylococcales
f__Staphylococcaceae
g__Jeotgalicoccus
s__Jeotgalicoccus_unclassified
o__Micrococcales
o__Actinomycetales
f__Actinomycetaceae
g__Flaviflexus
s__Flaviflexus_unclassified
g__Atopostipes
s__uncultured_Atopostipes_sp.
g__Aerococcus
s__Aerococcus_sp._148a
g__Jeotgalibaca
g__Alloiococcus
s__Alloiococcus_unclassified
o__Acholeplasmatales
f__Acholeplasmataceae
g__Anaeroplasma
s__Anaeroplasma_unclassified
s__Clostridium]_saccharolyticum
g__Eubacterium]_ruminantium_group
s__Eubacterium]_ruminantium_group_unclassified
0
2
4
LDA score
Group
DW4
CW4
CW0
BW4
AW4
AW0
